# Disruption of the glucagon receptor increases glucagon expression beyond α-cell hyperplasia in zebrafish

**DOI:** 10.1016/j.jbc.2022.102665

**Published:** 2022-11-09

**Authors:** Qi Kang, Jihong Zheng, Jianxin Jia, Ying Xu, Xuanxuan Bai, Xinhua Chen, Xiao-Kun Zhang, F. Susan Wong, Chao Zhang, Mingyu Li

**Affiliations:** 1 School of Pharmaceutical Sciences and School of Life Sciences, Xiamen University, Xiamen, China; 2 Fujian Provincial Key Laboratory of Innovative Drug Target Research, School of Pharmaceutical Sciences, Xiamen University, Xiamen, China; 3 Fundamental Research Center, Shanghai YangZhi Rehabilitation Hospital (Shanghai Sunshine Rehabilitation Center), School of Life Sciences and Technology, Tongji University, Shanghai, China; 4 Key Laboratory of Biotechnology of Fujian Province, Institute of Oceanology, Fujian Agriculture and Forestry University, Fuzhou, China; 5 Division of Infection and Immunity, Cardiff University School of Medicine, Cardiff, UK; 6 Department of Plastic and Reconstructive Surgery, Shanghai Ninth People's Hospital, Shanghai Jiao Tong University School of Medicine, Shanghai, China

**Keywords:** Glucagon receptor, Single-cell sequencing, Zebrafish, Glucagon, Pancreatic α-cells, DEG, differentially expressed gene, dpf, days postfertilization, GCGR, glucagon receptor, sgRNA, single-guide RNA, TEM, transmission electron microscopy, UMAP, uniform manifold approximation and projection

## Abstract

The glucagon receptor (GCGR) is a potential target for diabetes therapy. Several emerging GCGR antagonism-based therapies are under preclinical and clinical development. However, GCGR antagonism, as well as genetically engineered GCGR deficiency in animal models, are accompanied by α-cell hyperplasia and hyperglucagonemia, which may limit the application of GCGR antagonism. To better understand the physiological changes in α cells following GCGR disruption, we performed single cell sequencing of α cells isolated from control and *gcgr*^*−/−*^ (glucagon receptor deficient) zebrafish. Interestingly, beyond the α-cell hyperplasia, we also found that the expression of *gcga*, *gcgb*, *pnoca*, and several glucagon-regulatory transcription factors were dramatically increased in one cluster of *gcgr*^*−/−*^ α cells. We further confirmed that glucagon mRNA was upregulated in *gcgr*^*−/−*^ animals by *in situ* hybridization and that glucagon promoter activity was increased in *gcgr*^*−/−*^;*Tg(gcga:GFP)* reporter zebrafish. We also demonstrated that *gcgr*^*−/−*^ α cells had increased glucagon protein levels and increased granules after GCGR disruption. Intriguingly, the increased mRNA and protein levels could be suppressed by treatment with high-level glucose or knockdown of the *pnoca* gene. In conclusion, these data demonstrated that GCGR deficiency not only induced α-cell hyperplasia but also increased glucagon expression in α cells, findings which provide more information about physiological changes in α-cells when the GCGR is disrupted.

The hormone glucagon is a 29 amino acid polypeptide, which is predominantly secreted from the pancreatic α-cells. Glucagon is a major counter-regulatory hormone to insulin, and it plays a key role in glucose homeostasis by stimulating hepatic glucose production in the fasting state. Accumulating data suggest that diabetes is a “bi-hormonal” disease characterized by relative hypoinsulinemia and hyperglucagonemia, and the increased glucagon aggravates hyperglycemia by activating hepatic glucose production (1, 2).

Glucagon acts on the glucagon receptor (GCGR) (3), which is a seven-transmembrane–spanning G protein–coupled receptor that activates intracellular adenylate cyclase. This activation leads to increased intracellular cAMP, which mediates the metabolic pathways that include gluconeogenesis, glycolysis, and fatty acid oxidation (4). As glucagon signaling plays a central role in glucose homeostasis and contributes to diabetes pathophysiology, there has been considerable interest in targeting the GCGR for treatment of diabetes. Blockade of GCGR by small molecule antagonists, antisense molecules, or antibodies can improve glycemic control in both rodent diabetes models and humans with diabetes (5, 6, 7, 8). However, GCGR antagonism is usually accompanied by α-cell hyperplasia, lipid metabolism disorders, and hepatic fat accumulation (9, 10). In animal models, GCGR deficiency also results in α-cell hyperplasia, hyperglucagonemia, and hyperaminoacidemia (11, 12). Similar phenomena were also observed in humans with genetic mutation of the GCGR gene (13, 14).

Although the mechanism by which α-cell hyperplasia occurs has not been fully elucidated, disruption of GCGR has been shown to cause hepatic amino acid catabolic disorders and increased circulating amino acid levels, in particular L-glutamine and alanine, which then stimulate the α-cell hyperplasia through the amino acid transporter Slc38a5 (15, 16, 17, 18).

Hyperglucagonemia in GCGR-deficient animals may be attributable to the increased α-cell mass or increased glucagon secretion. Although there is no direct evidence that the hyperglucagonemia occurs because of α-cell hyperplasia in GCGR-deficient animals, it is possible that hyperplastic α-cells secrete more glucagon, which results in hyperglucagonemia. Although two studies have shown that expression and secretion of glucagon were increased during GCGR inhibition (19, 20), whole islet RNA-seq and whole pancreas were used, and the studies could not discern whether these increases were due to increase in the number of α-cells or due to individual cell boost. Thus far, no prior studies have determined whether α-cells boost glucagon expression or secretion in GCGR deficiency at the single cell level. In this study, we performed single-cell transcriptomic sequencing of α-cells from *gcgr*^*−/−*^ and control zebrafish. We found that a cluster of α-cells from the *gcgr*^*−/−*^ group dramatically increased the glucagon gene expression level compared to the control group. We further revealed that the α-cells in *gcgr*^*−/−*^ zebrafish increased glucagon protein and glucagon granules. These results suggested that α-cells increase glucagon expression when they become hyperplastic in *gcgr*^*−/−*^ zebrafish.

## Results

### Single-cell transcriptomic sequencing of α cells from GCGR KO zebrafish

**Figure 1:**
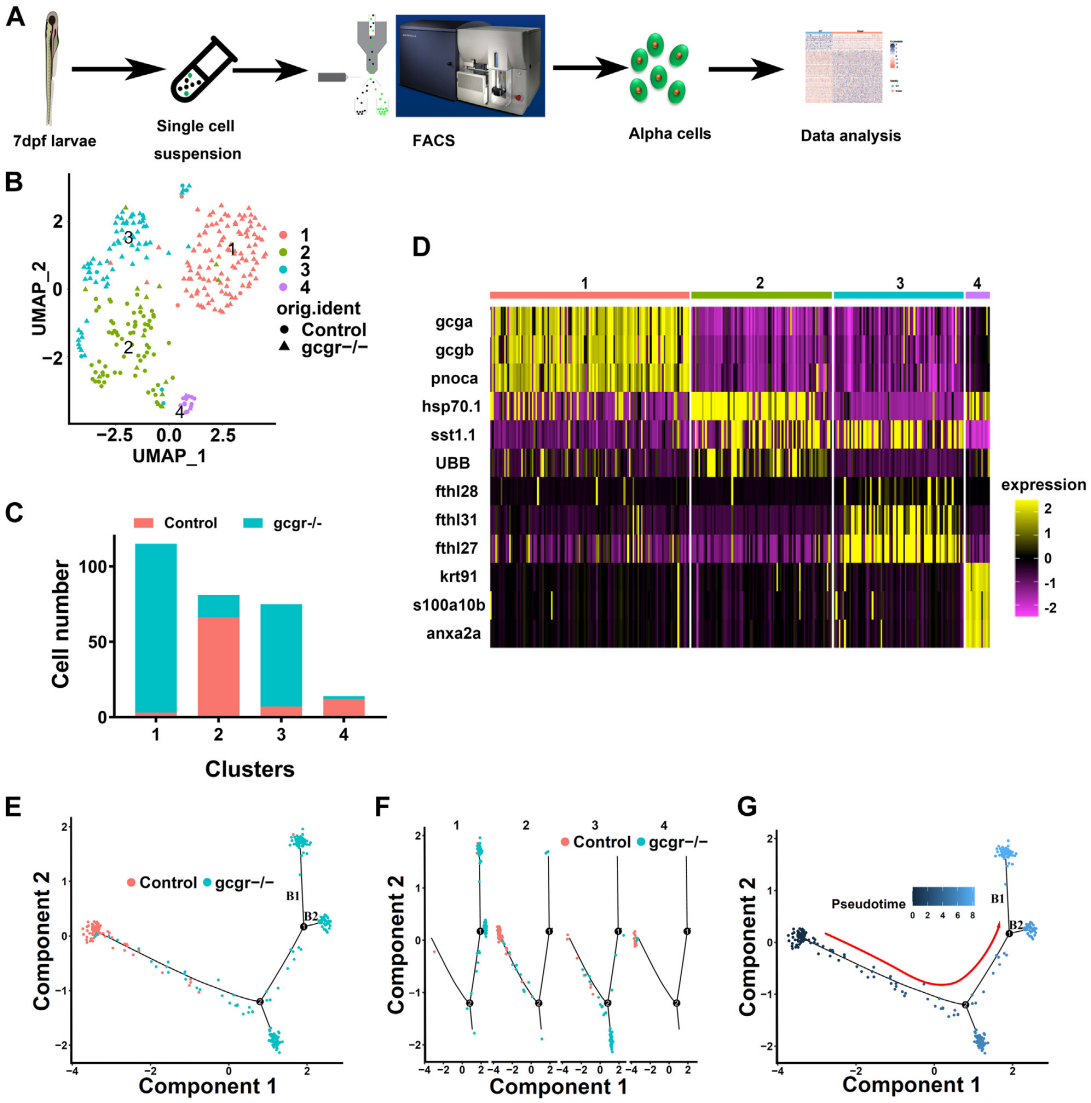
**The single-cell transcriptomic overview of αcells from *gcgr***^***−/−***^**and control zebrafish.***A*, schematic of single-cell isolation and single-cell RNA-seq from the zebrafish reporter line *Tg (gcga:GFP)*. *B*, distribution of all α cells by uniform manifold approximation and projection for dimension reduction (UMAP) analysis. All cells were segregated into four clusters, each cluster was labeled using different colors, the circles and triangles indicate α-cells from control and *gcgr*^*−/−*^, respectively. *C*. the cell numbers of each cluster 1 to 4 are shown. *D*, the heatmap shows the top 3 markers in each cluster; the color scale ranges from *magenta* to *yellow* corresponding to the gene expression as indicated. *E*–*G*, the pseudotime trajectory analysis of control and *gcgr*^*−/−*^ mutant α-cells. *E*, trajectory colored by samples of control or *gcgr*^*−/−*^. *F*, trajectory colored by individual clusters. *G*, trajectory colored by pseudotime, and the color in each cell depends on the pseudotime value ranging from 0 to 8. All plots for pseudotime trajectory are based on cell distribution as shown in Fig. 1*E*. All cells from control and *gcgr*^*−/−*^ mutant were aligned in pseudotime trajectory by monocle v2.16.0, number 1 and 2 presented decision point 1 and point 2; B1 and B2 presented branch 1 and branch 2.

GCGR blockade induced hyperglucagonemia and pancreatic α-cell hyperplasia, which suggested functional physiological changes in the α cells in response to the GCGR blockade (11, 17, 18). In order to investigate these changes, with high resolution, in *gcgr*^*−/−*^ zebrafish α cells in individual cell populations, we isolated single α cells from 7 days postfertilization (dpf) *Tg(gcga:GFP)* and *gcgr*^*−/−*^;*Tg(gcga:GFP)*. We extracted RNA from individual cells and performed single-cell transcriptomic sequencing using a modified Smart-seq2 approach (Fig. 1*A*) (21, 22). In total, data from 384 *gcgr*^*−/−*^ α cells and 192 control α cells were obtained from sequencing. After quality control, having excluded potential contaminating β cells and δ cells, 285 high-quality cells were obtained for subsequent analysis. A total of 24,900 genes were detected in these analyses. On average, 953 genes were detected with 28,914 counts sequenced per cell (Table S1). Cluster analysis divided the cells into four clusters, based on uniform manifold approximation and projection (UMAP) for dimension reduction (Fig. 1*B*). In clusters 1 and 3, most cells were from the *gcgr*^*−/−*^ group, whereas in clusters 2 and 4, most cells were from the control group (Fig. 1*C*, Table S2).

To characterize the clusters, we enriched genes for each cluster (Table S3, Fig. S1). For each of these clusters, the top 3 genes were as follows: cluster 1 (*gcga, gcgb* and *pnoca*), cluster 2 (*hsp70.1*, *sst1.1* and *UBB*), cluster 3 (*fthl28*, *fthl31* and *fthl27*), and cluster 4 (*krt91*, *s100a10b* and *anxa2a*) (Fig. 1*D*, and Table S3). We also performed a development trajectory analysis in an unbiased pseudotemporal manner for all of the 285 cells. Our results showed that the constructed trajectory comprised 4 branches and two decision points, and cells from control and *gcgr*^*−/−*^ showed different location preferences (Fig. 1, *E* and *F*). We further reconstructed a pseudotemporal trajectory by pseudotime value, which indicated the possible direction of the trajectory (Figs. 1*G* and S2). We then assessed the expression profile of 4 α-cell marker genes, *gcga*, *gcgb*, *arxa*, and *pax6b*, in pseudotime trajectory (Fig. S3). Our results indicated that cells located at the branches extended by decision point 1, expressed much higher levels of these marker genes. Since the majority of these cells located in branch B1 and branch B2 belonged to the *gcgr*^*−/−*^ groups, this demonstrated that the α cells from *gcgr*^*−/−*^ zebrafish were in a different physiological state compared with the control group. Taken together, these data suggested that knockout (KO) of GCGR in zebrafish resulted in transcriptional profile changes in α cells, and the α cells in *gcgr*^*−/−*^ zebrafish had cellular heterogeneity in response to the GCGR deficiency.

### KO of GCGR in zebrafish increased glucagon expression in α cells beyond α-cell hyperplasia

**Figure 2:**
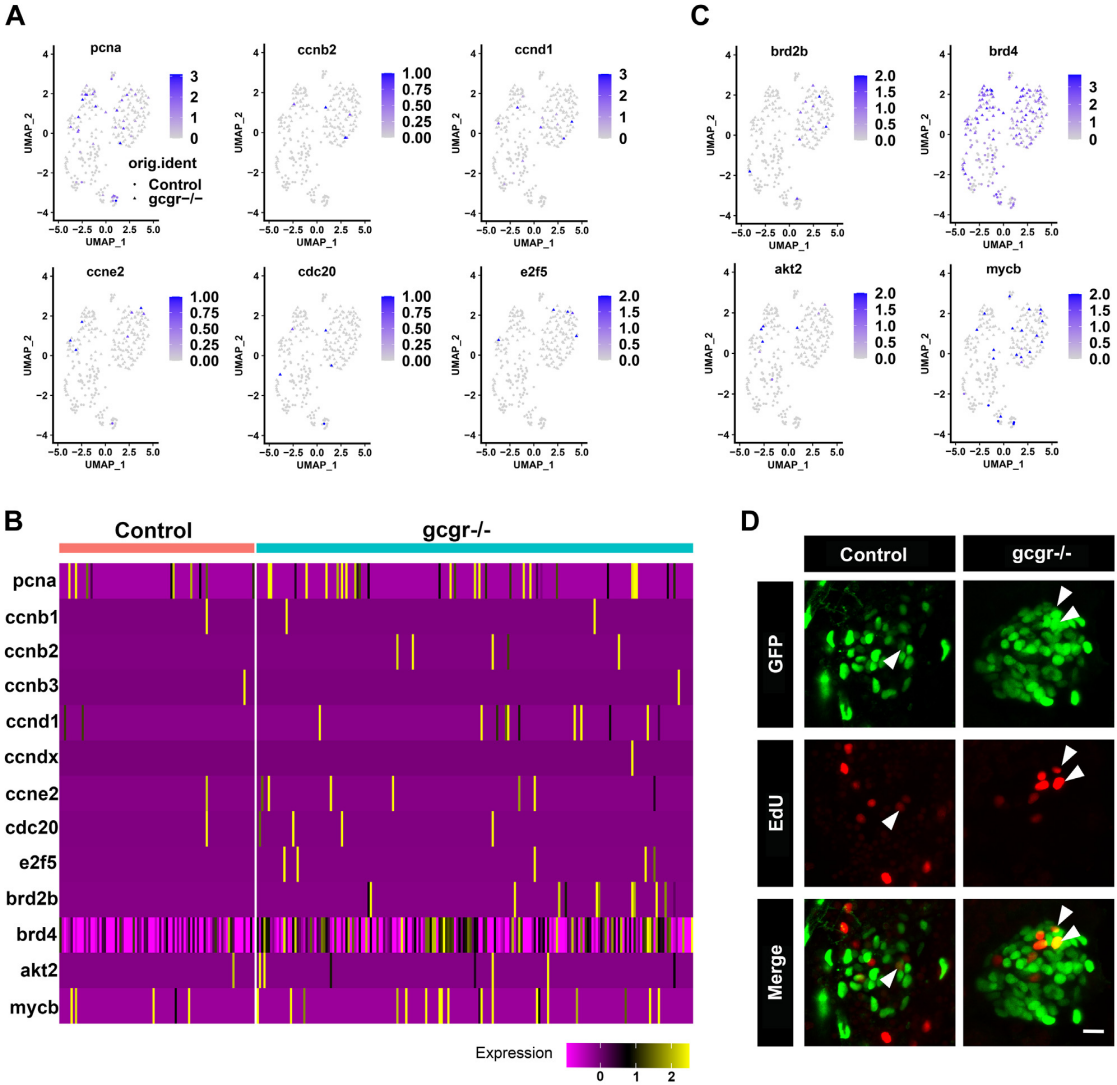
**KO of GCGR in zebrafish induced proliferation in αcells.***A*, the plots show the expression of several cell cycle regulatory genes in all cells, the circles and triangles indicate α cells from control and *gcgr*^*−/−*^ zebrafish, respectively, the color scale ranges *gray* to *blue* corresponding to expression level from low to high, all plots are based on cell clusters shown in Fig. 1*B*. *B*, heatmap of 13 cell cycle regulatory and associated genes in α cells from control and *gcgr*^*−/−*^ group. *C*, the plots show the expression of several cell cycle associated genes in all cells. The columns represent cells and the rows represent genes as indicated. The color scale ranges *purple* to *yellow* corresponding to expression level from low to high, all plots are based on cell clusters shown in Fig. 1*B*. *D*, representative images of EdU-labeled zebrafish islets from control *Tg(gcga:GFP)* and *gcgr*^*−/−*^*;Tg(gcgr*^*−/−*^*;gcga:GFP)* groups; the EdU^+^ and GFP^+^ cells represent the proliferative α-cells, which are indicated by the arrows; the scale bar represents 10um. GCGR, glucagon receptor.

KO of GCGR has been reported to increase α-cell hyperplasia in mouse and zebrafish (11, 23). We analyzed our RNA-seq data to examine the proliferating α cells. As shown in Fig. 2, *A* and *B*, the expression of several cell cycle regulators, including *pcna*, *ccnb2*, *ccnd1*, *ccne2*, *cdc20*, *e2f5*, are higher in the *gcgr*^*−/−*^ group compared with the control group. Similar trends were seen in some cell cycle–associated genes, for example, *brd2*, *brd4*, *akt2*, and *mycb* (Fig. 2, *B* and *C*). Moreover, the phenotype of increased proliferating cells in the *gcgr*^*−/−*^ group was again confirmed using an EdU staining approach (Fig. 2*D*). These data suggested that KO of GCGR increased the α-cell proliferation in zebrafish, in the small number of cells that we were able to test.

**Figure 3:**
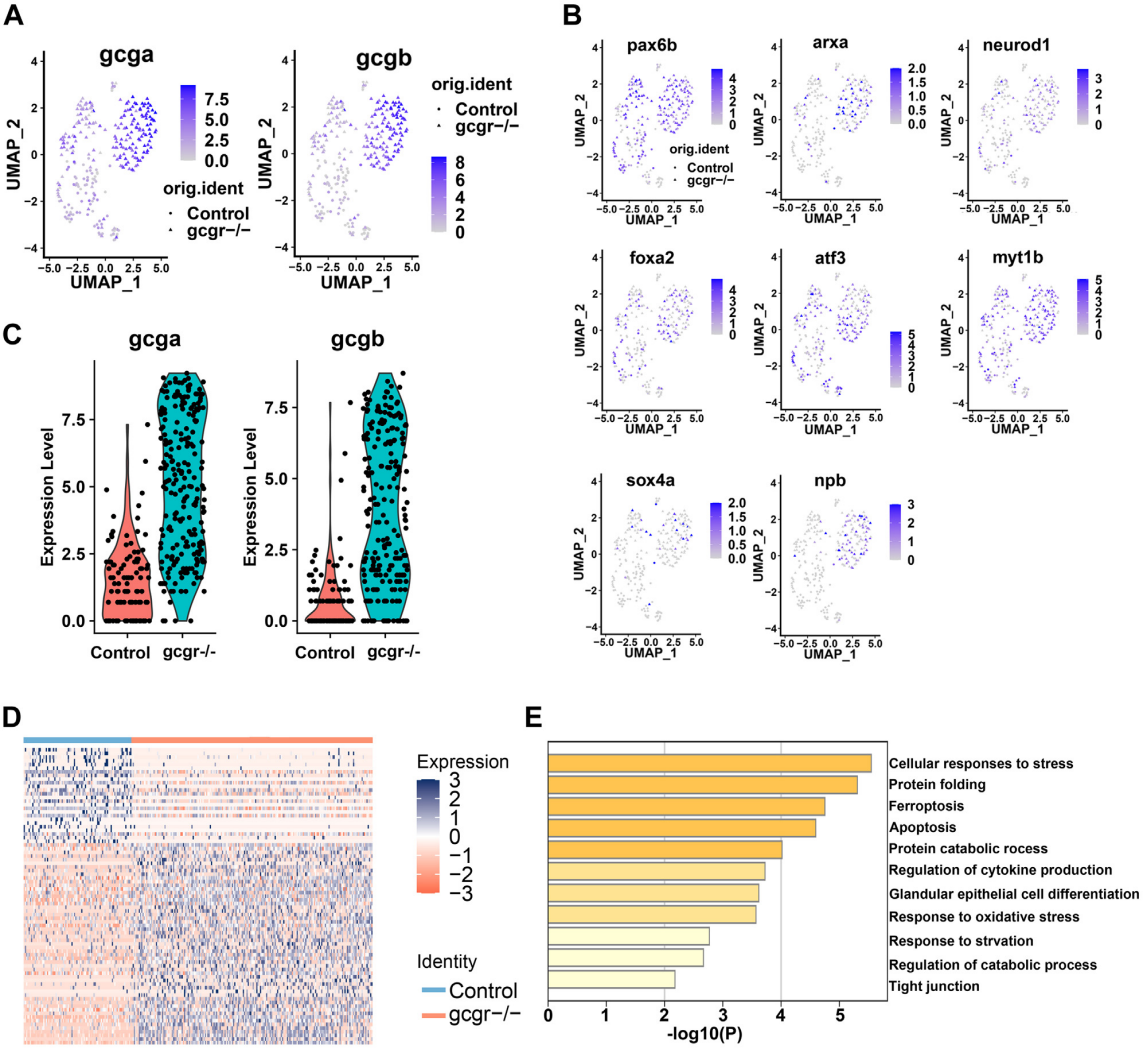
**KO of GCGR in zebrafish increased glucagon expression in α-cells.***A* and *B*, the plots show the expression of glucagon genes (*A*) and their regulators (*B*). The color scale ranges *gray* to *blue* corresponding to expression level from low to high; all plots are based on cell clusters shown in Fig. 1*B*. *C*, the violin plots show *gcga* and *gcgb* relative expression in α-cells from control and *gcgr*^*−/−*^ zebrafish. *D*, heatmap of differentially expressed genes (DEGs) of *gcgr*^*−/−*^ compared to control cells; the color scale ranges *red* to *blue* corresponding to expression level from low to high. *E*, pathway enrichment analysis of DEGs between *gcgr*^*−/−*^ and control cells. GCGR, glucagon receptor.

Subsequently, we then investigated other physiological changes in α cells in *gcgr*^*−/−*^ zebrafish at the single cell level. Given that 97% of α cells in cluster 1 were from *gcgr*^*−/−*^ zebrafish, which expressed glucagon genes (*gcga* and *gcgb*) at a high level (Fig. 3*A* and Fig. 1*C*), we then further analyzed this cluster 1. The regulation of glucagon gene expression is tightly controlled by a series of transcriptional factors (3, 24) and we found that *pax6b*, *arxa*, *neurod1*, *sox4a*, *foxa2*, *atf3*, *myt1b*, and *npb*, which are important for glucagon expression and α-cell function, were enriched in the cluster 1 α cells (Fig. 3*B*). These data suggested that the KO of GCGR in zebrafish increased the expression levels of glucagon genes and their transcriptional regulators in cluster 1 α cells.

To survey total transcriptional profile changes in *gcgr*^*−/−*^, we analyzed the differentially expressed genes (DEGs) between the control and *gcgr*^*−/−*^ groups (total α-cells) (Fig. S4 and Table S4). There was a considerable increase in expression levels with *gcga* 51.47-fold increased and *gcgb* 19.58-fold increased in the *gcgr*^*−/−*^ zebrafish (Fig. 3*C* and Table S4). We also found that *pax6b*, *arxa*, *neurod1*, *sox4a*, *foxa2*, *atf3*, *isl1*, *myt1b*, and *npb* were also increased in the *gcgr*^*−/−*^ α cells (Fig. S5). Furthermore, there were more upregulated than the downregulated genes in *gcgr*^*−/−*^ α cells (Fig. 3*D*). Gene annotation analysis of the DEGs revealed that genes involved in cellular responses to stress and protein folding were highly enriched (Fig. 3*E*).

**Figure 4:**
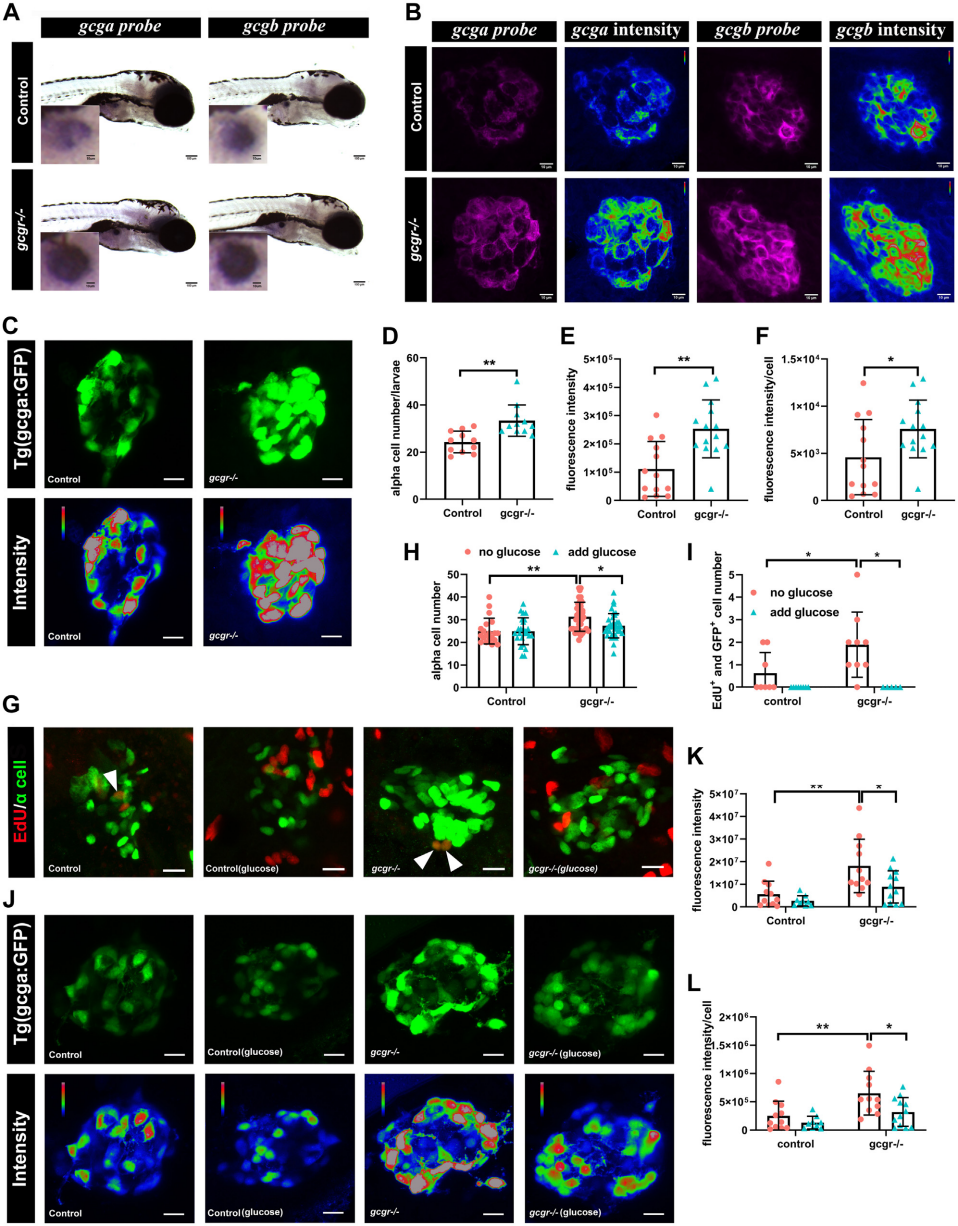
**Validation of single cell RNA data showing upregulated glucagon expression in *gcgr***^***−/−***^**zebrafish αcells.***A*, whole mount *in situ* hybridization analysis of *gcga* and *gcgb* in the control and *gcgr*^*−/−*^ α cells. The scale bar represents 100um, Scale bar in inset = 10um. *B*, fluorescence *in situ* hybridization analysis of *gcga* and *gcgb* in control and *gcgr*^*−/−*^ α cells. The scale bars represent 10 μm. *C*, representative images of the fluorescence (*green*) and intensity (*rainbow*) of α-cells from *Tg(gcga:GFP)* and *gcgr*^*−/−*^*;Tg(gcga:GFP)* zebrafish. The scale bars represent 10 μm. *D*, quantification of the α-cell number from *Tg(gcga:GFP)* and *gcgr*^*−/−*^*;Tg(gcga:GFP)* zebrafish. *E*, quantification of fluorescence intensity from *Tg(gcga:GFP)* and *gcgr*^*−/−*^*;Tg(gcga:GFP)* zebrafish. *F*, the average fluorescence intensity in each α-cell from *Tg(gcga:GFP)* and *gcgr*^*−/−*^*;Tg(gcga:GFP)* zebrafish. *D*–*F*, data represent mean ± SD with significance determined by unpaired two-tailed *t* test, ∗ *p*< 0.05; ∗∗ *p*< 0.01. *G*–*L*, hyperexpression and hyperplasia of glucagon in *gcgr*^*−/−*^ α-cells was inhibited by a high level of glucose. *G*–*I*, representative EdU staining images (*G*), α-cell quantification (*H*), and EdU-positive α-cell quantification (*I*) of different genotype larvae treated by high-level glucose. GFP+ and EdU+ cells represent the proliferative α-cells, which are indicated by arrows. Data represent mean ± SD with significance determined by two-way ANOVA, ∗ *p*< 0.05. The number of larvae (n) =20 to 36 for 4H, 5 to 8 for 4I. ANOVA results for 4H: interaction: F (1, 112) = 3.047, *p* = 0.0836; Row factor (control *versus gcgr*^*−/−*^): F (1, 112) = 15.16, *p* = 0.0002; Column factor (glucose treatment): F (1, 112) = 3.141, *p* = 0.0791. ANOVA results for 4I: interaction: F (1, 26) = 3.252, *p* = 0.083; Row factor (control *versus gcgr*^*−/−*^): F (1, 26) = 3.252, *p* = 0.083; Column factor (glucose treatment): F (1, 26) = 12.86, *p* = 0.0014. *J*, representative images of the fluorescence (*green*) and intensity (*rainbow*) of α-cells from *Tg(gcga:GFP)*, *gcgr*^*−/−*^*;Tg(gcga:GFP)* zebrafish, and high glucose-treated *Tg(gcga:GFP)* and *gcgr*^*−/−*^*;Tg(gcga:GFP)* zebrafish. The images are confocal projections; the scale bars represent 10 μm. *K*–*L*, quantification of total fluorescence intensity of GFP (*K*), and average fluorescence intensity of GFP per cell (*L*) from *Tg(gcga:GFP)*, *gcgr*^*−/−*^*;Tg(gcga:GFP)* zebrafish, and high glucose-treated *Tg(gcga:GFP)* and *gcgr*^*−/−*^*;Tg(gcga:GFP)* zebrafish. Data represent mean ± SD with significance determined by two-way ANOVA, ∗ *p*< 0.05; ∗∗ *p*< 0.01, ∗∗∗*p* < 0.001. The number of larvae (n) =9 to 11 ANOVA results for 4K: interaction: F (1, 39) = 1.789, *p* = 0.1887; Row factor (control *versus gcgr*^*−/−*^): F (1, 39) = 15.60, *p* = 0.0003; Column factor (glucose treatment): F (1, 39) = 6.641, *p* = 0.0139. ANOVA results for 4L: interaction: F (1, 39) = 1.553, *p* = 0.2201; Row factor (control *versus gcgr*^*−/−*^): F (1, 39) = 12.00, *p* = 0.0013; Column factor (glucose treatment): F (1, 39) = 7.076, *p* = 0.0113.

To further validate the high transcriptional level of glucagon genes in *gcgr*^*−/−*^ mutants identified from single-cell sequencing data, we then performed whole-mount *in situ* hybridization, both in light and fluorescence approaches, using *gcga* and *gcgb* probes. Our data showed that both *gcga* mRNA and *gcgb* mRNA increased in the *gcgr*^*−/−*^ α cells (Fig. 4, *A* and *B*). Furthermore, we employed the α-cell reporter line *Tg(gcga:GFP)*, which used zebrafish glucagon promoter to drive GFP-specific expression in the α cells (25), for further analysis. Seven days postfertilization, WT and *gcgr*^*−/−*^ larvae were fixed and the pancreatic islet area imaged under the confocal microscope using identical capture criteria. α-cell numbers and α-cell fluorescence intensity were measured (Fig. 4*C*). Similar to our previous data (23), the α-cell numbers were increased in *gcgr*^*−/−*^ mutants (22.5 ± 1.3 *versus* 31.8 ± 1.9) (Fig. 4*D*). The total fluorescence intensity per islet was increased in *gcgr*^*−/−*^ mutants (Fig. 4*E*). Strikingly, the average fluorescence intensity of single α cell was significantly higher in *gcgr*^*−/−*^ mutants compared with the controls (Fig. 4*F*). Taken together, these data further suggested that the KO of GCGR in zebrafish upregulated glucagon gene expression in α cells, as well as induced α-cell hyperplasia.

Our previous studies revealed that the total free glucose in *gcgr*^*−/−*^ zebrafish was lower than in the WT control, and a high level of glucose suppressed α-cell hyperplasia in *gcgr*^*−/−*^ zebrafish (12, 23). Hence, we further investigated whether the high level of glucose affected the glucagon expression in *gcgr*^*−/−*^ and control α cells in this study. When we coincubated *gcgr*^*−/−*^*;Tg(gcga:GFP)* and *Tg(gcga:GFP)* with high levels of glucose (20 mM), the α-cell number and α-cell proliferation were decreased in *gcgr*^*−/−*^ zebrafish (Fig. 4, G–I). Interestingly, the total GFP fluorescence intensity of α cells and average fluorescence intensity per α cell in *gcgr*^*−/−*^ zebrafish was significantly reduced (Fig. 4, *J*–*L*). These data suggested that high-level glucose suppressed the glucagon expression and inhibited the α-cell proliferation in *gcgr*^*−/−*^ zebrafish.

### KO of GCGR in zebrafish increases glucagon protein and granule populations in α cells

**Figure 5:**
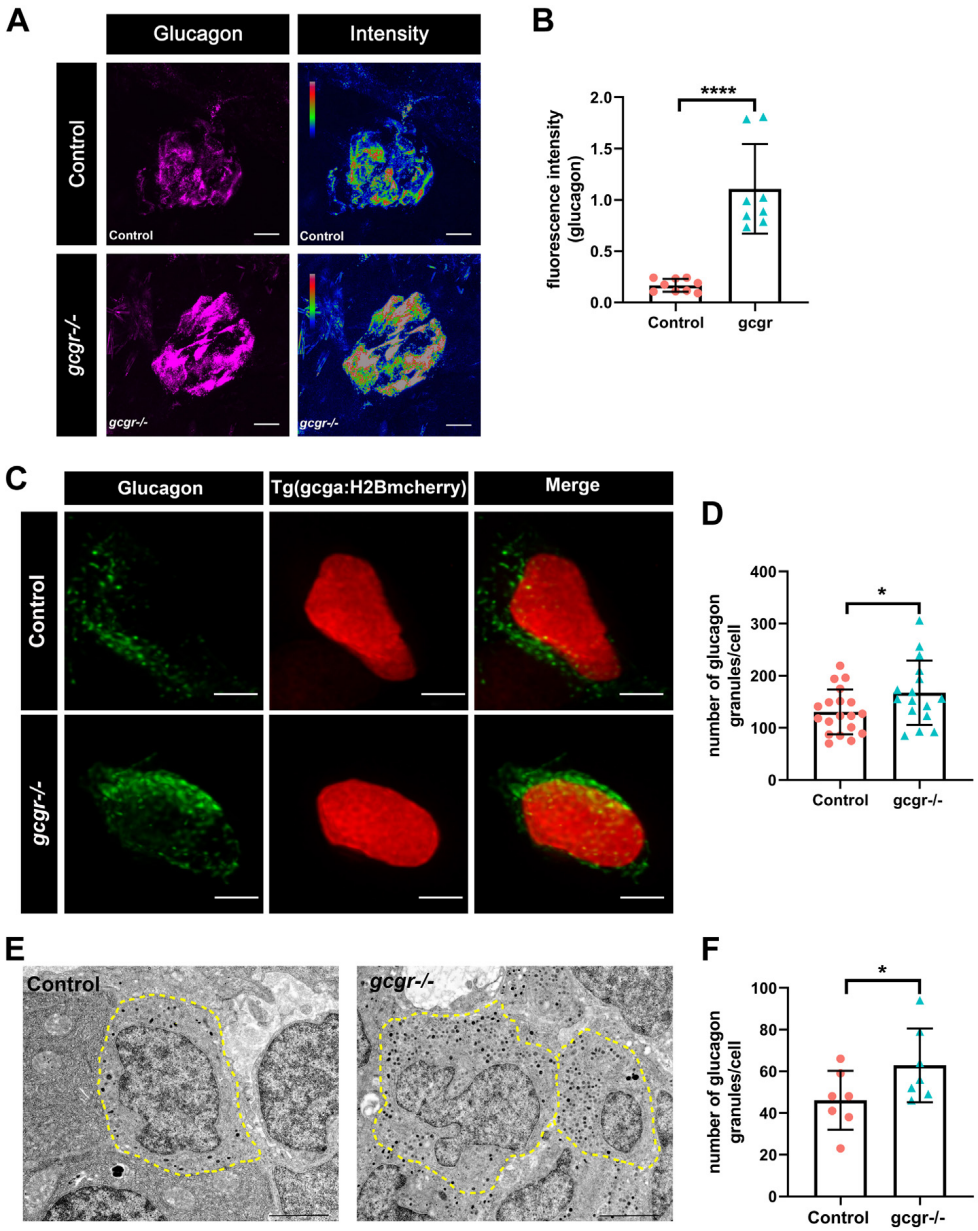
***gcgr***^***−/−***^**zebrafish increase glucagon granules and glucagon level.***A*, representative images of the fluorescence (*magenta*) and intensity (*rainbow*) of α-cells from control and *gcgr*^*−/−*^ zebrafish, which are indicated by immunostaining with antiglucagon antibody; the scale bar represents 10um. *B*, quantification of fluorescence intensity of the images from control and *gcgr*^*−/−*^ zebrafish. Data represent mean ± SD with significance determined by unpaired two-tailed *t* test, ∗∗∗∗*p* < 0.0001. The number of larvae (n) = 8 to 9. *C*, representative images of α-cells in *Tg(gcga:H2BmCherry)* and *gcgr*^*−/−*^;*Tg(gcga:H2BmCherry)* larvae, immunostained with antiglucagon antibody. *Green* staining indicates intracellular glucagon granules; mCherry protein shown as *red* labels the nucleus of α cells. The scale bar represent 2 μm. *D*, quantification of the glucagon granules content per cell from immunostained *Tg(gcga:H2BmCherry)* and *gcgr*^*−/−*^;*Tg(gcga:H2BmCherry)* zebrafish. Data represent mean ± SD with significance determined by unpaired two-tailed *t* test, ∗ *p*< 0.05, n =16 to 19 cells. *E*, electron micrographs of control and *gcgr*^*−/−*^ zebrafish. The cell boundary is depicted by dotted lines, and the glucagon granules are as *gray* and *black* dots in the cells. *F*, quantification of the glucagon granules content of control and *gcgr*^*−/−*^ from transmission electron microscopy (TEM). The scale bar represents 2 μm. Data represent mean ± SD with significance determined by unpaired two-tailed *t* test, ∗ *p*< 0.05, n = 15 to 18 cells.

We further investigated whether the high-level expression of glucagon genes in the *gcgr*^*−/−*^ zebrafish had commensurate effects on glucagon protein levels and glucagon granules. Firstly, we performed immunostaining in control and *gcgr*^*−/−*^ zebrafish by using antiglucagon antibody. As shown in Fig. 5, *A* and *B*, the fluorescence intensity of the glucagon signal was upregulated in *gcgr*^*−/−*^ α cells, which indicated elevated glucagon protein levels. We also assessed the effect of high-level glucose on glucagon protein levels in *gcgr*^*−/−*^ α cells and observed that at a high level of glucose (20 mM), the fluorescence intensity of the glucagon signal was significantly reduced in the *gcgr*^*−/−*^ zebrafish (Fig. S6).

To analyze the glucagon granules, a *Tg(gcga:H2BmCherry)* reporter line, which specifically labeled the nuclei of α cells with mCherry, was employed here. Seven days post fertilization, *gcgr*^*−/−*^;*Tg(gcga:H2BmCherry)* and control *Tg(gcga:H2BmCherry)* were stained with antiglucagon antibody, and the glucagon granule population was examined under the confocal microscope using the same criteria. As shown in Fig. 5, *C* and *D*, *gcgr*^*−/−*^ mutants significantly increased the number of glucagon granules in their α cells. To further investigate these changes in numbers of glucagon granules in *gcgr*^*−/−*^, we performed ultrastructural analysis of the α cells in *gcgr*^*−/−*^ and WT zebrafish larvae using transmission electron microscopy (TEM). Similar to the immunostaining analysis results, the TEM results indicated that the glucagon granule population was significantly increased in *gcgr*^*−/−*^ larvae (Fig. 5, *E* and *F*). Taken together, these data revealed that KO of GCGR led to an increase in glucagon protein and the glucagon granule population in zebrafish α cells.

### Pnoca plays an important role in the regulation of α-cell function in gcgr^−/−^ zebrafish

**Figure 6:**
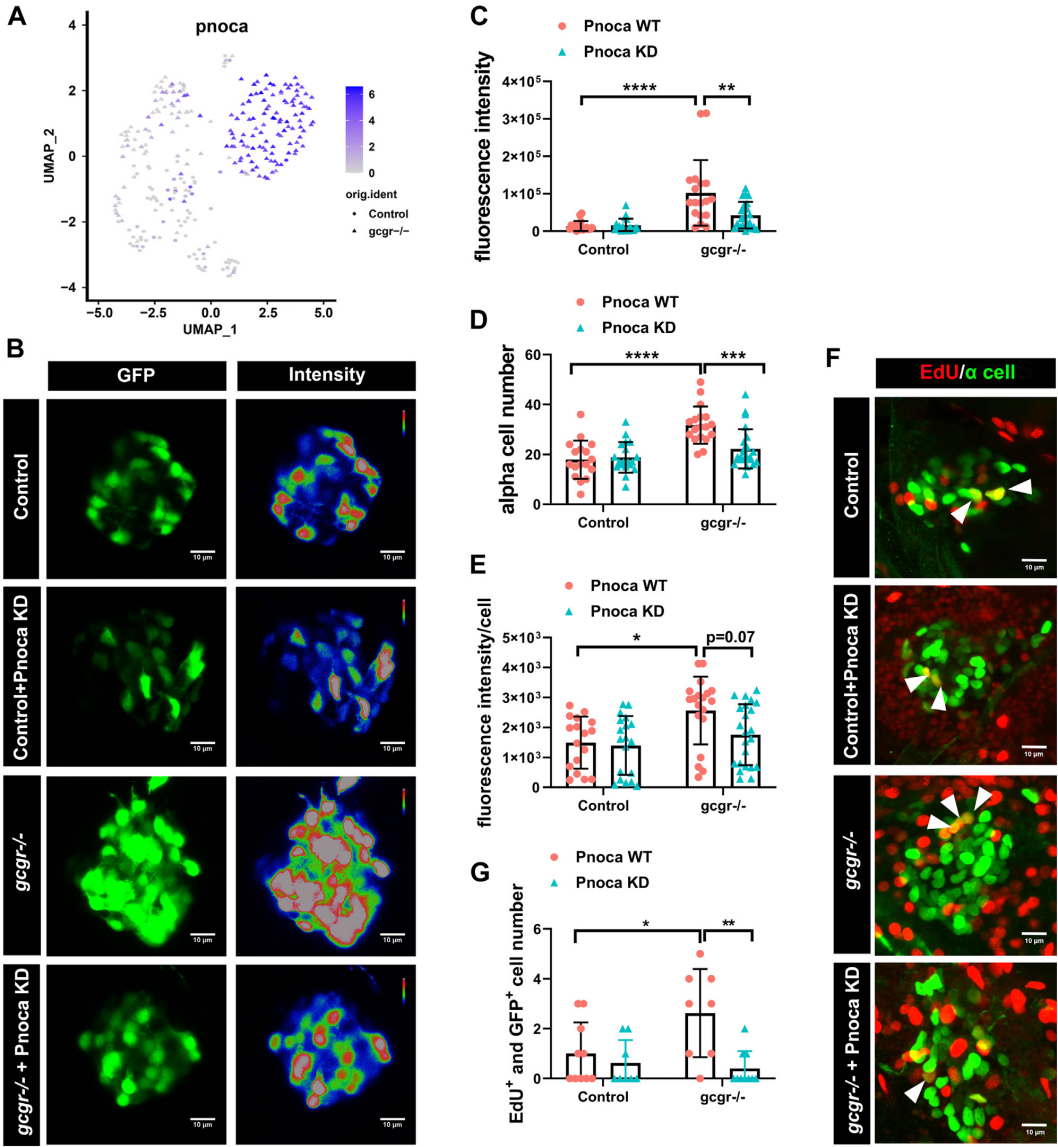
**Knockdown of *pnoca* reduces α-cell number and glucagon expression in the *gcgr***^***−/−***^**zebrafish group.***A*, the plots show the expression of *pnoca* gene, all plots are based on cell clusters shown in Fig. 1*B*. *B*, representative images of the fluorescence intensity of α cells from *Tg(gcga:GFP)*(control), *pnoca* knockdown (pnoca KD) *Tg(gcga:GFP)*, *gcgr*^*−/−*^*;Tg(gcga:GFP)*, and *pnoca* knockdown *gcgr*^*−/−*^*;Tg(gcga:GFP)* zebrafish in their respective groups. *C*–*E*, quantification of the islet fluorescence intensity (*C*) α-cell number (*D*) and fluorescence intensity in per cell (*E*) from control, *gcgr*^*−/−*^, and *pnoca* knockdown in the respective group. Data represent mean ± SD with significance determined by two-way ANOVA, ∗ *p*< 0.05; ∗∗ *p*< 0.01, ∗∗∗ *p*< 0.001, ∗∗∗∗ *p* < 0.0001, n =17 to 23. ANOVA results for 6C: interaction: F (1, 73) = 7.829, *p* = 0.0066; Row factor (control *versus gcgr*^*−/−*^): F (1, 73) = 27.97, *p* = 0.0001; Column factor (*pnoca* knockdown): F (1, 73) = 6.847, *p* = 0.0108. ANOVA results for 6D: interaction: F (1, 73) = 9.566, *p* = 0.0028; Row factor (control *versus gcgr*^*−/−*^): F (1, 73) = 26.31, *p* = 0.0001; Column factor (*pnoca* knockdown): F (1, 73) = 6.523, *p* = 0.0127. ANOVA results for 6E: interaction: F (1, 74) = 2.425, *p* = 0.1237; Row factor (control *versus gcgr*^*−/−*^): F (1, 74) = 9.723, *p* = 0.0026; Column factor (*pnoca* knockdown): F (1, 74) = 3.856, *p* = 0.0533. *F*–*G*, the representative EdU staining images (*F*) and quantification (*G*) of *Tg(gcga:GFP)*(control), *pnoca* knockdown (pnoca KD) *Tg(gcga:GFP)*, *gcgr*^*−/−*^*;Tg(gcga:GFP)*, and *pnoca* knockdown *gcgr*^*−/−*^*;Tg(gcga:GFP)* zebrafish. GFP+ and EdU+ cells represent the proliferative α cells, which are indicated by arrows. *G*, data represent mean ± SD with significance determined by two-way ANOVA ∗ *p* < 0.05; ∗∗ *p* < 0.01, n=8 to 10. ANOVA results for 6G: interaction: F (1, 32) = 5.274, *p* = 0.0283; Row factor (control *versus gcgr*^*−/−*^): F (1, 32) = 3.020, *p* = 0.0919; Column factor (*pnoca* knockdown): F (1, 32) = 10.42, *p* = 0.0029.

Further investigating enriched genes in *gcgr*^*−/−*^ α cells, *pnoca* (which encodes prepronociceptin) was increased 19.36 fold (Figs. 1*D*, 6A and Table S4), and it is specifically expressed in zebrafish α cells in the islet (26). We examined the role of *pnoca* in the regulation of α-cell function during GCGR deficiency. We performed a knockdown of *pnoca* by coinjecting two single-guide RNA (sgRNA) and Cas9 proteins into embryos at the one-cell stage and then examined the efficiency of mutagenesis individually at 7 dpf. The successful knockdown larvae were processed for further analysis. As shown in Fig. 6, *B*–*E*, knockdown of *pnoca* reduced both α-cell numbers and α-cell fluorescence intensity (per cell) in *gcgr*^*−/−*^*;Tg(gcga:GFP)* larvae. Knockdown of *pnoca* did not affect β-cell number (Fig. S7). Moreover, the decreased α-cell numbers in *gcgr*^*−/−*^ zebrafish was mainly due to suppressed α-cell proliferation but not increased α-cell death (Figs. 6, *F*–*G*, and S8). However, knockdown of *pnoca* neither affected α-cell numbers nor α-cell fluorescence intensity in the *Tg(gcga:GFP)* control group. Taken together, these data suggested that *pnoca* plays an important role in the regulation of α-cell function in *gcgr*^*−/−*^ zebrafish, especially for glucagon expression and α-cell proliferation.

## Discussion

In recent years, investigators have recognized that glucagon and the hormonal effects of glucagon play a central role in the pathogenesis of type 2 diabetes (1, 27). Approaches using GCGR antagonism and GCGR KO rodent animals have shown promising effects on glucoregulation in both type 1 and 2 diabetes (28, 29, 30, 31). However, antagonizing the effects of glucagon when used as a possible therapeutic could have unwanted effects. The obvious side effects are hyperglucagonemia and α-cell hyperplasia. In animal models, the glucagon signal blockade leads to a dramatic increase in the circulatory glucagon level (11, 23, 32). A similar phenomenon was observed in patients with spontaneous glucagon receptor gene mutations or in clinical trials using antiglucagon receptor monoclonal antibody (13, 33, 34). The compensatory excessive glucagon production may limit the application of GCGR antagonism for diabetes therapy. To clarify the mechanism of GCGR blockade-induced hyperglucagonemia, most studies have focused on the α-cell hyperplasia (15, 17, 18, 35). However, no studies have gained deep insight into glucagon expression and secretion in the α-cells.

Thus, in our study, we examined effects of removal of the glucagon receptor on the α cells producing glucagon and glucagon homeostasis. We took advantage of the genetic tractability of zebrafish and recently developed single-cell RNA-seq technology. In the zebrafish GCGR KO model *gcgr*^*−/−*^, we found a small population of proliferating α cells (Fig. 2). In addition, we demonstrated that α cells in *gcgr*^*−/−*^ zebrafish dramatically increased glucagon (both *gcga* and *gcgb*) expression, as well as increased several transcription factors that regulate glucagon expression by single-cell RNA-seq analysis (Fig. 3). We further showed that glucagon mRNA was upregulated in *gcgr*^*−/−*^ animals and that *gcgr*^*−/−*^ α cells increased glucagon at the protein level, with increased glucagon granules after GCGR disruption (Figure 4, Figure 5). This could be physiologically regulated, as this increase could be suppressed by treatment with high glucose. All these data demonstrated that disruption of GCGR in zebrafish increased glucagon mRNA and protein levels beyond α-cell hyperplasia.

We also found that prepronociceptin a (*pnoca*) was highly upregulated in the α cells of *gcgr*^*−/−*^ zebrafish (Figs. 1*D* and 6A). Knockdown of *pnoca* in *gcgr*^*−/−*^ animals significantly reduced glucagon expression, as well as decreased the α-cell numbers due to suppression of proliferation (Fig. 6). Recently, *pnoca* was identified as a novel marker in zebrafish α cells (26). In addition, *pnoca* was dramatically downregulated when *pax6b,* a key regulator of pancreatic endocrine cells differentiation (36), was inactivated. However, prepronociceptin (*PNOC*) mRNA was barely detectable in α cells of mice and humans (Fig. S9). Instead, *PNOC* mRNA was expressed widely throughout the brain in mice and humans (37, 38, 39). In mammalian cells, PNOC works as a large precursor protein, which can be proteolytically processed to generate multiple neuropeptides, including nociceptin/orphanin FQ (N/OFQ), nocistatin, and orphanin FQ2 (OFQ2). N/OFQ is a 17-amino acid neuropeptide that binds to the nociceptin receptor (NOP) and is involved in the regulation of pain sensitivity, thermoregulation, hyperphagia, and reward behavior (40, 41). These data suggested that the role of *pnoca* in α cells is probably a species-specific effect. Nevertheless, the function of *pnoca,* in zebrafish α cells still requires further elucidation.

**Figure 7:**
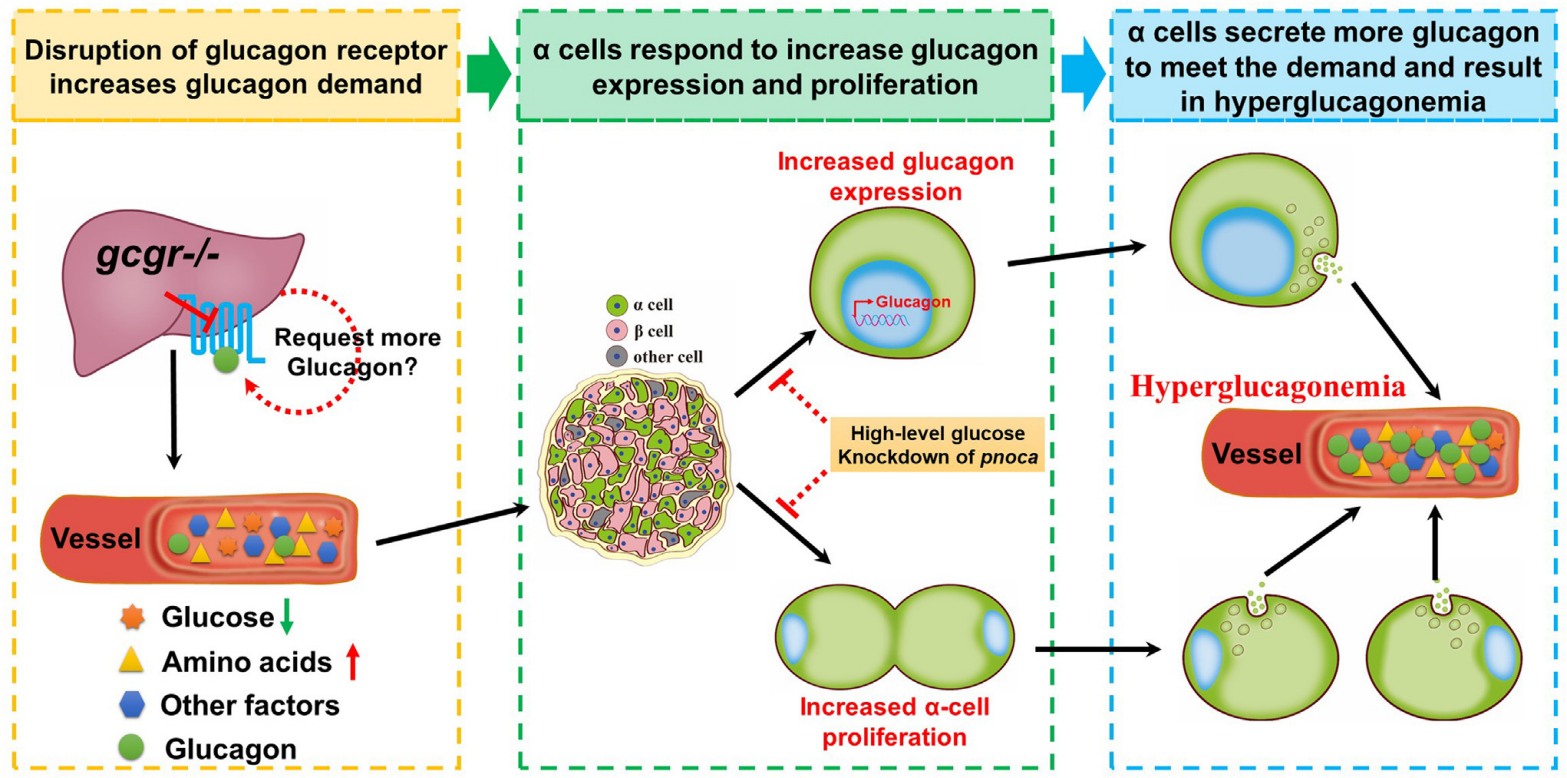
**A working model for GCGR disruption resulting in hyperglucagonemia.** Disruption of GCGR increases glucagon demand due to feedback signals from the liver and other tissues. The metabolic remodeling following GCGR disruption in the liver induces circulating factor/factors and these are transmitted to the islet. The α cells in the islet respond to these factors in two ways, one of which is to upregulate glucagon expression and the other is to induce α-cell hyperplasia. Both induce more glucagon secretion to meet the increased glucagon demand, which results in hyperglucagonemia in the circulation. However, administering high-level glucose or knockdown of *pnoca* in the GCGR KO animals suppresses both glucagon expression and α-cell hyperplasia. The dashed lines and circles indicate that there is uncertainty as to whether these effects are direct or indirect. GCGR, glucagon receptor.

Our study revealed that KO of GCGR resulted in increased glucagon expression in α cells, together with α-cell hyperplasia. Based on our results in this study and findings from our and other groups, we suggest a new working model of α-cell response during GCGR deficiency (Fig. 7). Once the GCGR is impaired, the glucagon-GCGR pathway is disrupted, which increases glucagon demand due to compensatory mechanisms. In response to the glucagon demand, α cells stimulated by factor/factors originating from the liver, such as amino acids, cause α-cell hyperplasia through proliferation (15, 16, 17, 18, 23, 42, 43). At the same time, the existing α cells also upregulate the glucagon mRNA and protein levels to meet the increased glucagon demand (this study). Both hyperplasia and increased glucagon expression in α cells lead to more glucagon secretion and in GCGR-disrupted animals resulted in hyperglucagonemia. Our study thus has provided more information about physiological changes in α cells during GCGR disruption. However, the detailed mechanisms by which α cells in GCGR-deficient animal increase glucagon expression need to be further elucidated. This will require investigation of other genes in the affected pathways including *pnoca*. Moreover, although the zebrafish islet shares physiological similarities with the mammalian islet, some aspects of physiological functional regulation in α cells may differ between zebrafish and mammalian models. Hence, whether these findings are recapitulated in the mouse and humans need to be further investigated.

## Experimental procedures

### Zebrafish line and maintenance

Zebrafish (*Danio rerio*) were maintained in a recirculating aquaculture system (Shanghai Haisheng Biotech Co, Ltd) with a 14 h:10 h light/dark cycle at 28 °C. The embryos were generated by natural breeding in the tank in an E3 embryo rearing solution at 28.5 °C and staged according to a standard protocol (44). In the research, *gcgra*^*−/−*^*;gcgrb*^*−/−*^ double mutant fish (referred as *gcgr*^*−/−*^ henceforth) (23), *Tg(gcga:GFP)* (25) and *Tg(gcga:H2bmCherry)* (45) were used. All procedures have been approved by the Xiamen University Institutional Animal Care and Use Committee (Protocol XMULAC20160089, 10 March 2016).

### Zebrafish islet isolation and single-cell suspension preparation for fluorescence-activated cell sorting

Seven days postfertilization, *Tg(gcga:GFP)* and *gcgr*^*−/−*^*;Tg(gcga:GFP)* zebrafish larvae were sacrificed and digested gently with collagenase P (1.2 mg/ml, Sigma–Aldrich) for 5 min at 37 °C, then the islets were hand-picked using forceps, from the homogenate under a fluorescence microscope (Lecia M205 FCA, Lecia Wetzlar). The isolated islets were further digested to a single cell suspension with Liberase DH (100 μg/ml, Roche) for 50 min at 36 °C. Finally, the GFP-positive cells were collected by fluorescence-activated cell sorting (BD AriaIII) into 96-well plate containing lysis buffer.

### Single-cell RNA-seq library preparation and sequencing

Single-cell 3′ end libraries were generated using a modified Smart-seq2 protocol. Single cells were collected into a 96-well plate containing reverse transcription buffer and incubated at 72 °C for 5 min, then the plate was chilled on ice. The oligo (dT) primer in reverse transcription buffer contained the oligo sequence for template switching and 9 bp unique molecular identifiers with an additional 8 bp cell barcode. Fifteen microliters PCR amplification mixture (5 μM ISPCR Primer, 7.5 mM dNTP mixture, 0.5U KAPA HiFi HotStart, 1× KAPA buffer, 12.5 mM MgCl2) was added to each well. The samples were PCR amplified (Bio-Rad C1000 Touch Thermal Cycler) using the following protocol: denaturation at 98 °C for 3 min, 24 cycles of (98 °C for 20 s, 67 °C for 15 s, and 72 °C for 6 min), with a final extension at 72 °C for 5 min. 1*X* AMPure XP bead (Beckman, A63882) and Qubit dsDNA HS Assay Kit (Invitrogen, Q32854) were used to purify the products from PCR amplification. A Nextera XT DNA sample kit (Illumina) was used to prepare the single cell libraries according to the manufacturer’s instructions. Single cell libraries were sequenced on an Illumina HiSeqX10 instrument generating paired-end 150-bp reads.

### Sequence alignment and gene expression matrix generation

The tail poly T of sequencing reads were first removed with customized scripts. The adapter in reads was trimmed using Trim Galore (-q 25 --phred33 --length 10 --stringency 3). Trimmed reads were aligned to the reads to the GRCz11 reference genome (UCSC) using STAR (46). The reads in each cell were counted using featureCounts (47) (-T 5 -p -t CDS). The gene expression matrix was generated by combining the expression of each cell. Finally, we obtained a matrix of 24,900 genes and 576 cells (384 *gcgr*^*−/−*^ α cells and 192 control α cells).

### Processing of single-cell RNA-seq data

Seurat R package v3.0 (48, 49) was used to perform further analysis including quality control, highly variable gene identification, dimensionality reduction, unsupervised clustering, and DEG identification. One-hundred thirty-four low-quality cells were removed if the detected gene number was lower than 500 or the proportion of mitochondrial genes greater than 10%. After quality filtering, the mean and median numbers of detected genes per cell were 1019.2 and 956.0, respectively. SCTransform function was used to normalize the counts and identify the highly variable genes. ElbowPlot function was applied to choose significant principal components (PCAs). Finally, 3000 highly variable genes and 30 PCAs were used as an input of dimensionality reduction. FindClusters function (reduction.type = ‘umap’, resolution= 0.8) was performed to cluster cells. For data visualization, we projected the cells in 2D space using UMAP.

### Differentially expressed markers identification and pathway enrichment analysis

FindAllmarkers function (min.pct = 0.25, logfc.threshold = 0.25, test.use=‘wilcox’) was conducted to find markers differentially expressed among clusters. FindMarkers function (min.pct = 0.25, logfc.threshold = 0.1, test.use=‘wilcox’, ident.1=‘*gcgr*^*−/−*^’, ident.2=‘control’) was performed to identify markers differentially expressed among *gcgr*^*−/−*^ α-cells and control α-cells. The website MetaScape (50) was used to perform the pathway enrichment analysis of gene clusters.

### Single-cell trajectory analysis

Monocle v2.16.0 (51) R package was used for single cell trajectory analysis. The DEGs between *gcgr*^*−/−*^ α-cells and control α-cells were used as “ordering genes.” A DDRTree method in “reduceDimension” function was chosen to reduce the dimensionality of the data. The “orderCells” function was used to order the cells along the trajectory.

### Whole-mount *in situ* hybridization and whole-mount immunofluorescence

The digoxigenin-labeled antisense RNA probes were synthesized *in vitro* by T3 RNA polymerase (Beyotime D7066). The whole-mount *in situ* hybridization was performed as described previously (23).

The whole-mount immunofluorescence was carried out as described by Jennifer *et al*. (52). The immunofluorescence was performed following *in situ* hybridization. In brief, the larvae were incubated with antiglucagon antibody (Sigma G2654) at 4 °C overnight, then washed with PBST (0.1% tween-20 in PBS). Alexa Fluor 488 secondary antibody (Thermo Fisher A11001, 1：1000) was used for detection following incubation for 2 h at room temperature (RT). The images were captured by Leica SP8 confocal system.

### Zebrafish α-cell imaging and fluorescence intensity measurement

The *Tg(gcga:GFP)* zebrafish larvae or immunofluorescence-stained larvae were fixed in 4% paraformaldehyde in PBS overnight and then flat mounted in Aqua-Mount (Richard-Allan Scientific) with their right side facing the coverslip. All the images were collected using a confocal microscope (Lecia Wetzlar, SP8). In addition, all the images were captured under identical criteria. Fluorescence intensity was analyzed by ImageJ software (National Institutes of Health). The total fluorescence value was analyzed based on a constant threshold. The representative rainbow images show the different intensity range in the different groups. The α-cell numbers of islets were counted manually based on the captured images. The intensity per cell was calculated using the following equation: intensity/cell= total intensity of the frame/total number of cells in the same frame.

### TEM analysis for zebrafish α cells

Seven days postfertilization, zebrafish larvae were fixed in 2% glutaraldehyde and 2% paraformaldehyde in 0.1 M sodium PBS for 2 h at RT. The islets were hand-picked under a stereoscopic fluorescence microscope (Leica, M205 FCA), mounted in 1% agarose, and then refixed in 1% osmium tetroxide in 0.1 M sodium PBS at RT for 2 h, followed by dehydration using gradient ethanol. Finally, the specimens were embedded in epoxy resin. The ultrathin sections were cut and mounted on copper grids; the sections were stained using uranyl acetate and lead citrate. The picture was captured by H-7800 transmission electron microscope (HITACHI). Zebrafish α cells (glucagon-producing) were identified by the presence of round dense core vesicles without any halo. In contrast, zebrafish β cells (insulin-producing) are characterized by the presence of immature secretory granules with light core as well as mature secretory granules with a dense core and a prominent halo.

### Inactivation of pnoca by Crispr-Cas9

For the *pnoca* knockdown, two sgRNA targets were designed: CGTGGTGTGACTGCCAGAAGG and GTGTCTGGTTGAGTGTCATGG. The sgRNAs were synthesized using an *in vitro* transcription kit (Invitrogen, AM1314). The mixture of two sgRNAs (50 ng/μl) and Cas9 protein (2 μM, NEB, M0646) was injected in fertilized zebrafish eggs. At 7 dfp, the efficiency of mutagenesis was tested by heteroduplex migration assays after PCR amplification as described previously (53). The mutagenesis ratio of zooids were analysis by ImageJ; the larvae with a mutagenesis ratio of more than 50% were used for further analysis.

### Glucose exposure

The *Tg(gcga:GFP)* and *gcgr*^*−/−*^*; Tg(gcga:GFP)* zebrafish larvae were incubated with 20 mM glucose in a 0.3× Danieau solution from 4 to 7 dpf for 3 days. At 7 dpf, the larvae were harvested and fixed in a 4% paraformaldehyde in PBS overnight for analysis.

### EdU staining

The proliferating α-cells were identified by the Click-iT EdU Alexa Fluor 594 Imaging Kit (C10339; Invitrogen). Six days postfertilization, zebrafish larvae were incubated with 1 mM 5-ethynyl-2-deoxyuridine (EdU) for 24 h in each group. EdU was detected by Click reaction according to the manufacturer’s protocol, and after the EdU staining, GFP (ProteinTech 50430-2-AP) immunostaining was performed to clarify the α cells. Images were captured by Leica SP8 microscope.

### Apoptosis analysis by TUNEL assay

For the cell apoptosis, we used a TUNEL assay. In brief, the PFA-fixed 7 dpf zebrafish larvae in each group were permeabilized using 10 μg/ml proteinase K for 30 min, and the apoptotic cells were stained using a TUNEL apoptosis detection kit Alexa Fluor 640 (YEASEN, 40308ES20) according to the manufacturer’s instructions. Images were captured by Leica SP8 microscope.

### Statistical analysis

Data are presented as means ± SD. Data were analyzed by two-way ANOVA for two-factor assays, followed by Tukey’s post hoc test, with all ANOVA analysis results reported in the figure legends. Two-tailed unpaired *t*-tests were used for single factor assays. Data were considered statistically significant at *p* < 0.05. Data analyses were performed using the GraphPad Prism 9 (GraphPad Software Inc).

## Data availability

All sequencing data were deposited in the Gene Expression Omnibus (GEO) repository (accession number GSE179894; https://www.ncbi.nlm.nih.gov/geo/query/acc.cgi?acc=GSE179894). The zebrafish strains used in this study are available from the corresponding author upon reasonable request.

## Ethics approval and consent to participate

All animal procedures conformed to the institutional guidelines of the Xiamen University Institutional Animal Care and Use Committee (IACUC).

## Supporting information

This article contains supporting information.

## Conflict of interest

The authors declare that they have no conflicts interest with the contents of this article.
