# Supplementary material for: Disruption of the glucagon receptor increases glucagon expression beyond α-cell hyperplasia in zebrafish
Source: J Biol Chem. 2022 Nov 9;298(12):102665. doi: 10.1016/j.jbc.2022.102665 (PMC9719020; doi:10.1016/j.jbc.2022.102665)
Supplement: Supplemental Figure [file mmc5.pdf]

Supplemental figures and figure legends

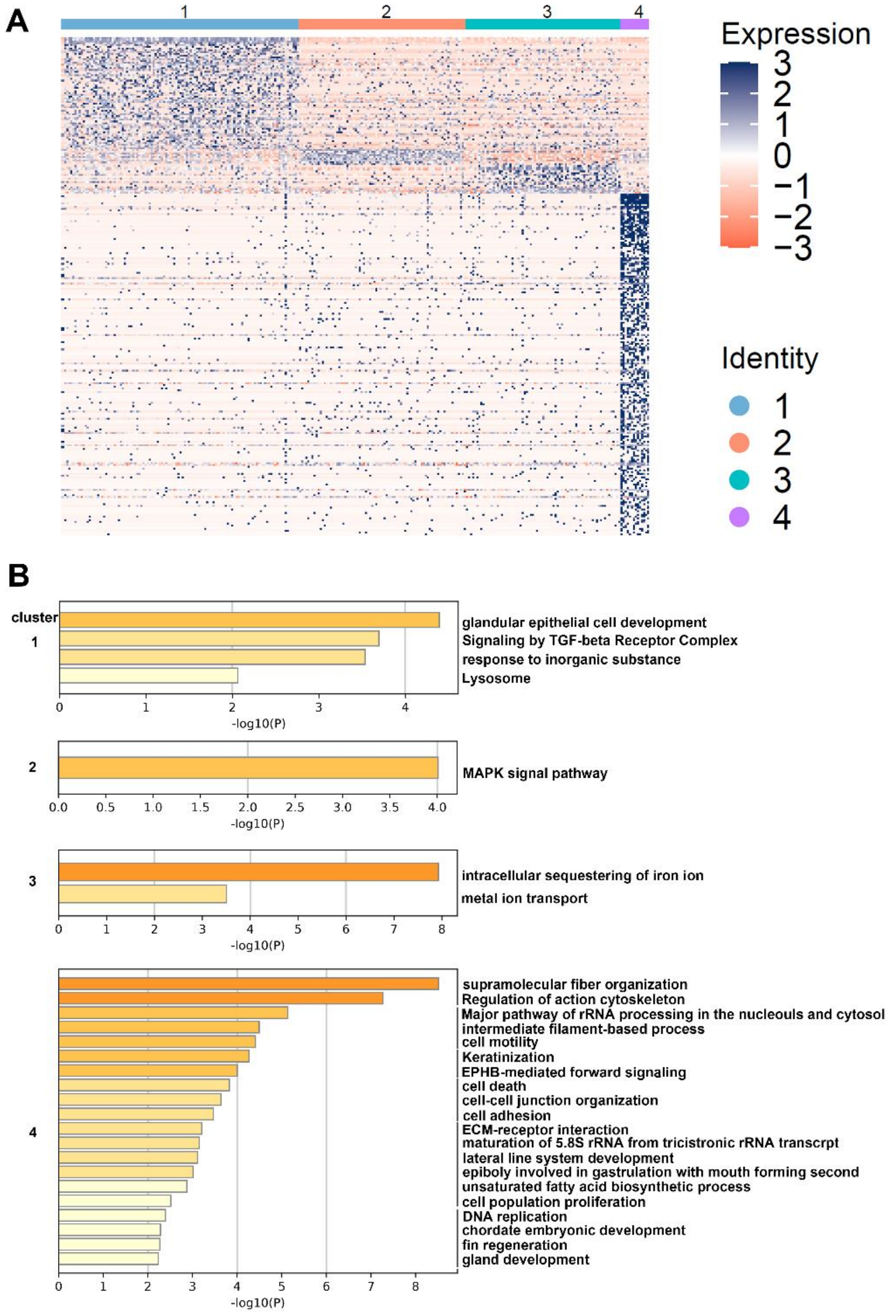

Figure S1, Analysis of the most variable genes in each cluster.

A, Heatmap of the highly expressed genes in each cluster. Each column represents a single cell sample, and each row represents one gene. The cell clusters were labeled on the top of the heatmap, the color scale ranges from orange to blue corresponding to low to high expression levels. **B**, Pathway enrichment analysis of the highly expressed genes in each cluster, the cell clusters are labeled on the left of the image, and the pathways are labeled on the right.

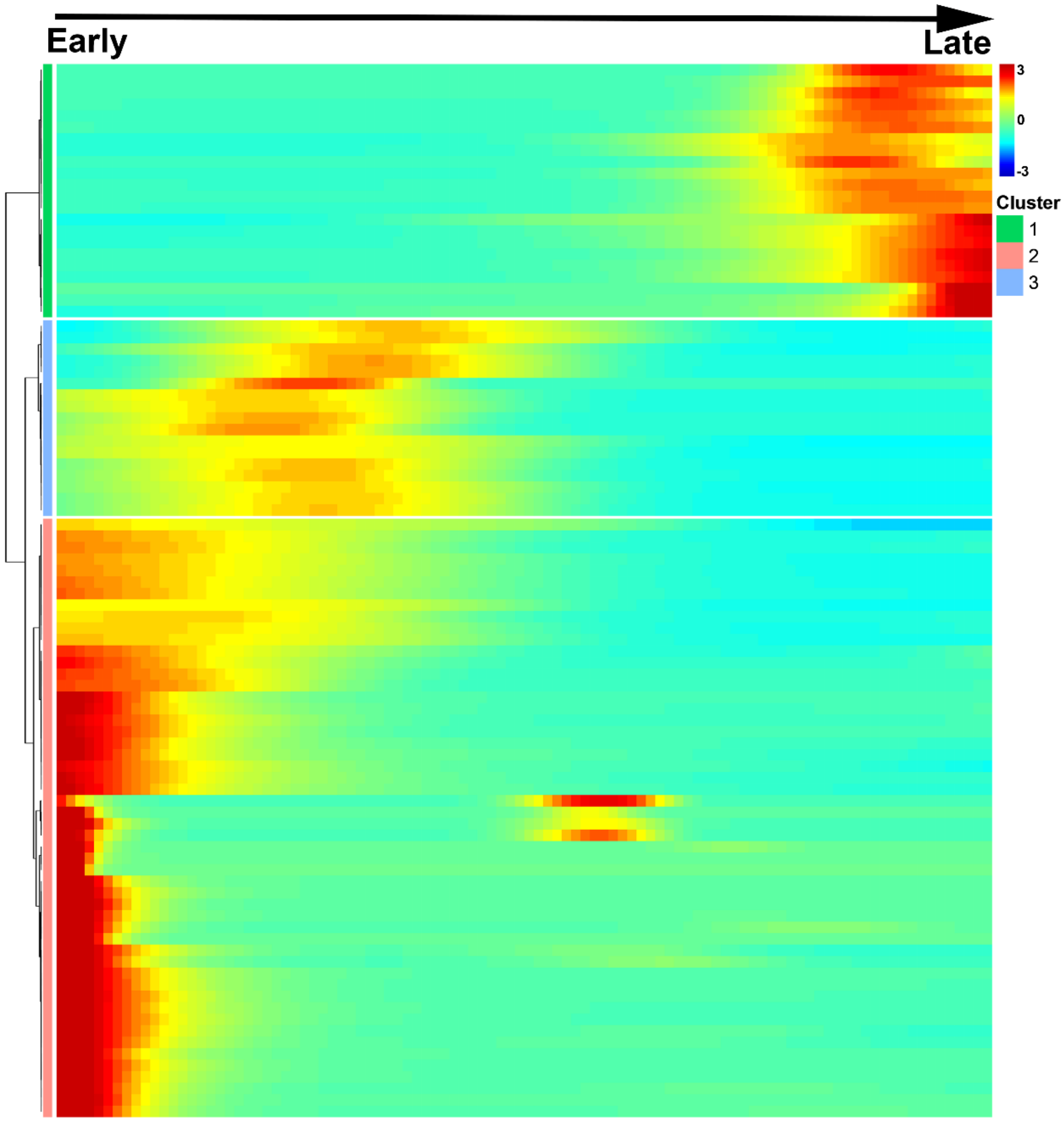

Figure S2, The dynamically expression genes across pseudotime.

The dynamically expression genes ($q<0.01$) are shown in the branched heat map. Each row represents a gene, and the color scheme represents the z-score distribution from -3.0 (blue) to 3.0 (red). The three cluster genes are shown as the early (cluster2), middle (cluster3), late (cluster1) expression patterns across pseudotime.

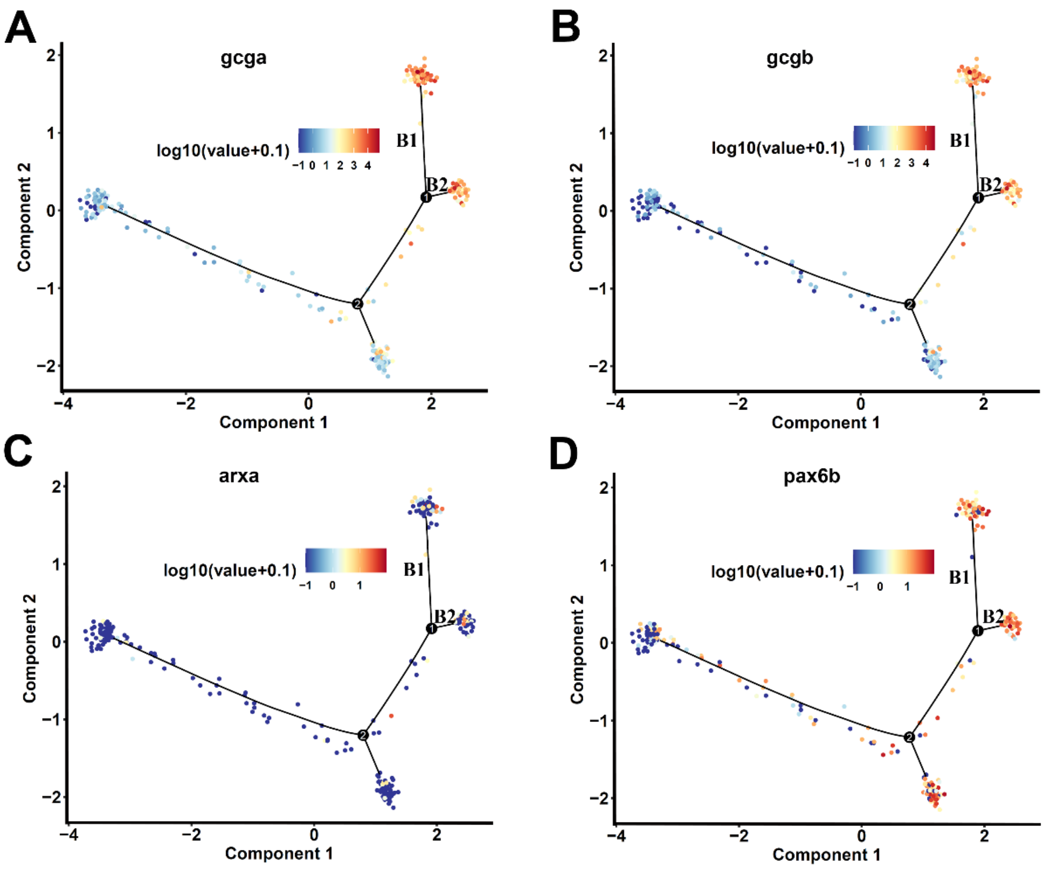

Figure S3, The α cell marker genes expression in pseudotime trajectory.

The expression tendency of α cell marker genes in pseudotime trajectory, *gcga* (**A**), *gcgb* (**B**), *arxa* (**C**) and *pax6b* (**D**). Numbers 1 and 2 represent decision point 1 and point 2; B1 and B2 represent branch 1 and branch 2. The color indicates the expression level in each cell, all plots for pseudotime trajectory are based on cell distribution as shown in Fig 1E.

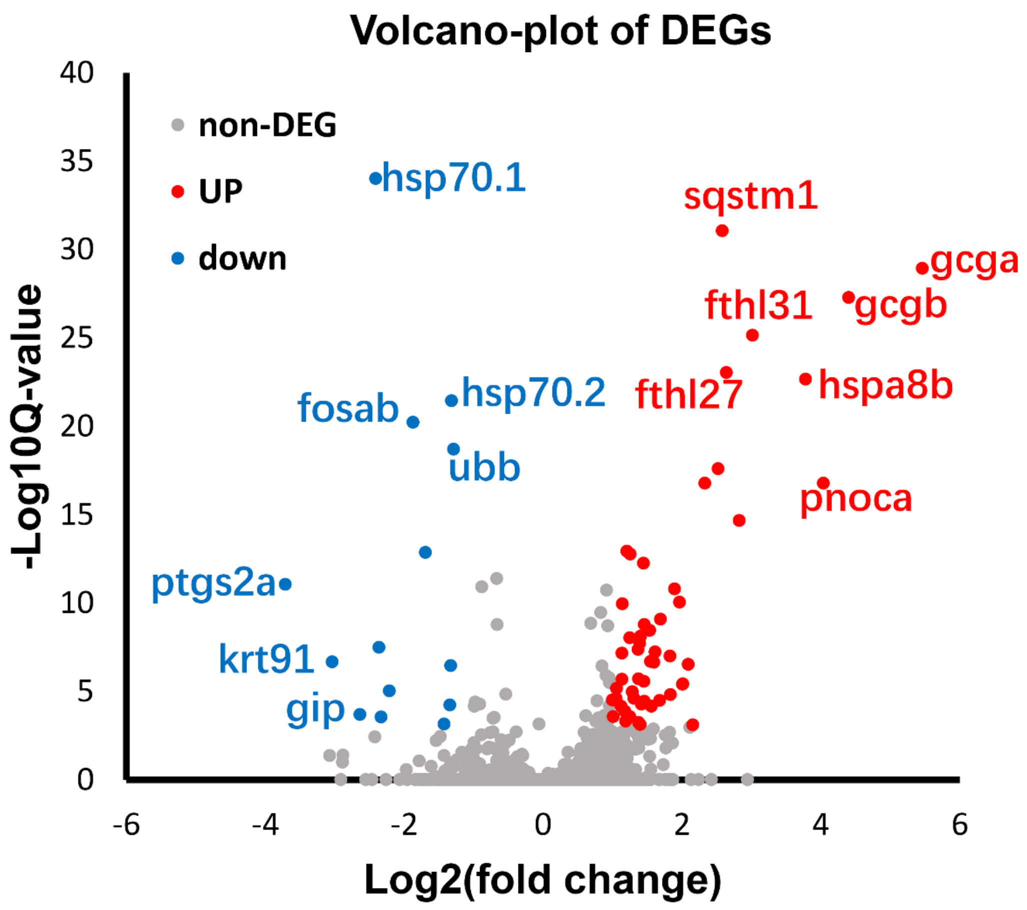

Figure S4, The volcano plot of DEGs (differentially expressed genes) between control and *gcgr*$^{-/-}$ zebrafish.

The DEGs are shown in the volcanic plot by the distribution of significance [-log10(q-value)] vs fold change [log2(fold change)] for genes. The up and down-regulated genes are labeled in red and blue respectively, and some DEGs are marked with the gene names.

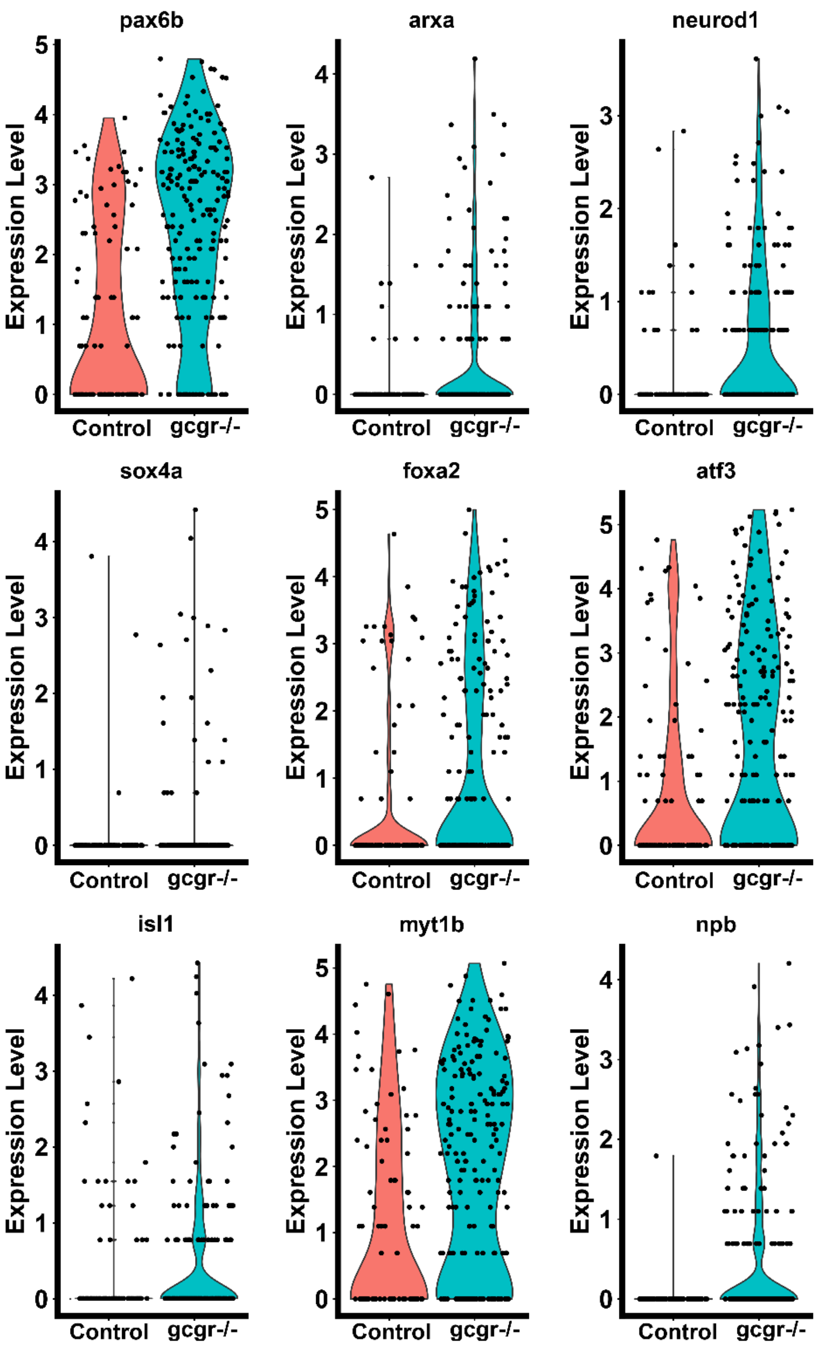

Figure S5, Violin plot for glucagon regulators compared between control and *gcgr*$^{-/-}$ zebrafish.

The violin plots show the relative expression of *pax6b*, *arxa*, *neurod1*, *sox4a*, *foxa2*, *atf3*, *isl1*, *myt1b* and *npb* in α cells from control (red) and *gcgr*$^{-/-}$ (green). The gene name is indicated on top of each violin plot and the value on the Y-axis represents the gene expression level in log1p(counts).

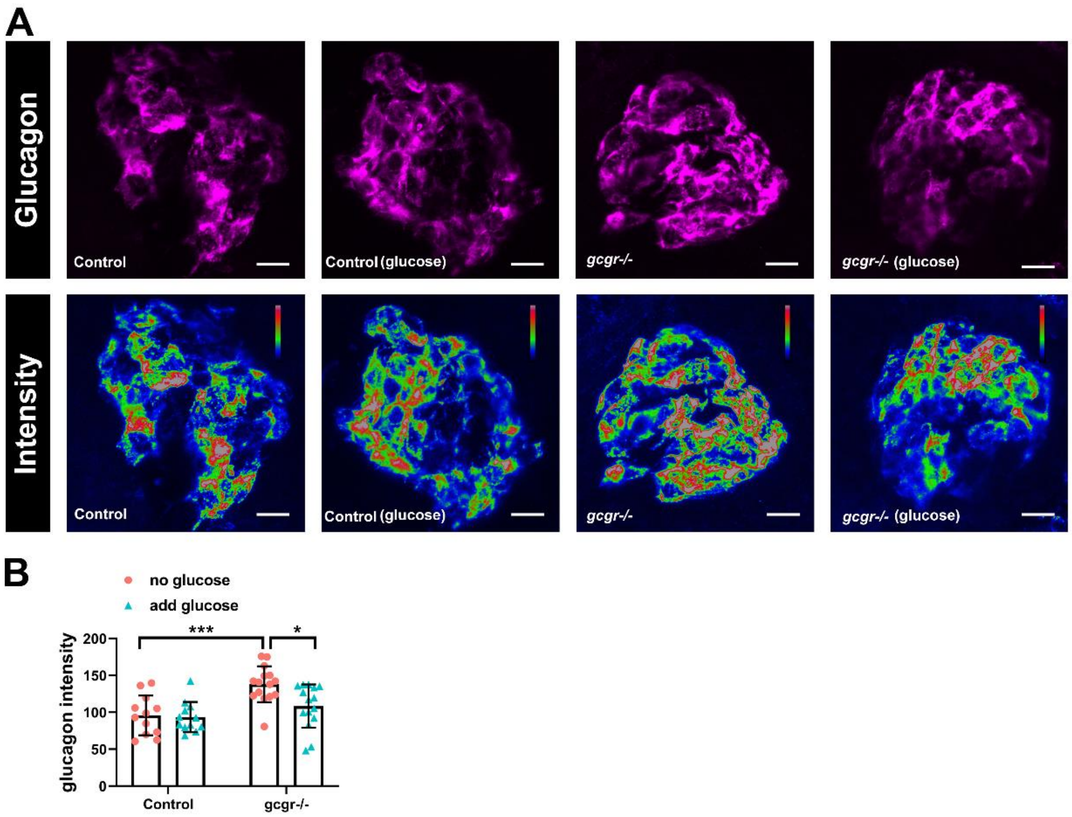

Figure S6, High-level glucose decreased the glucagon protein level in *gcgr*$^{-/-}$ α cells

A, Representative images of the fluorescence (magenta) and intensity (rainbow) of α cells from control, *gcgr*$^{-/-}$, and high-level glucose-treated control and *gcgr*$^{-/-}$ zebrafish, which are indicated by immunostaining with anti-glucagon antibody, scale bar = 10um. B, Quantification of fluorescence intensity of larvae from control, *gcgr*$^{-/-}$ zebrafish, and high-level glucose-treated control and *gcgr*$^{-/-}$ zebrafish. Data represent mean ± SD with significance determined by two-way ANOVA, * $p< 0.05$; *** $p< 0.001$, n=12-15. ANOVA results for S6B: interaction: $F (1, 50) = 3.771$, $p = 0.0578$; Row factor (control *vs gcgr*$^{-/-}$): $F (1, 50) = 16.52$, $p = 0.0002$; Column factor (glucose treatment): $F (1, 50) = 5.183$, $p = 0.0271$.

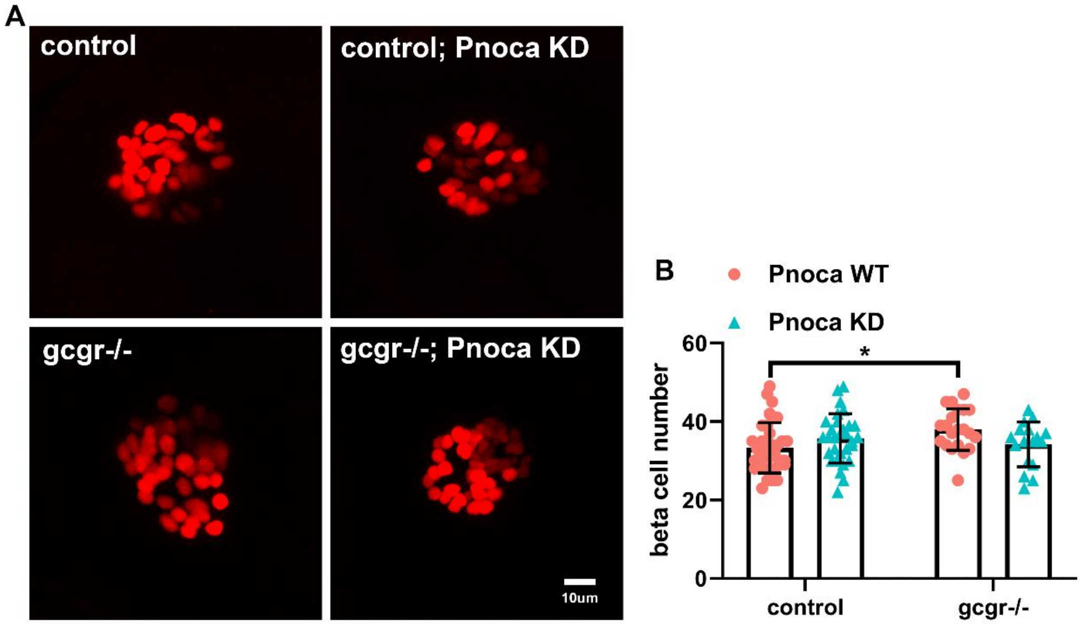

Fig S7, Knockdown of *pnoca* did not change the β-cell number in control and *gcgr*-/- zebrafish.

A, Representative images of control *Tg(ins:H2BmCherry)*, *pnoca*-knockdown *Tg(ins:H2BmCherry), gcgr*$^{-/-}$; *Tg(ins:H2BmCherry) and* pnoca-knockdown *gcgr*$^{-/-}$; *Tg(ins:H2BmCherry)* zebrafish. **B**, Quantification of the β cells in each group. Data represent mean ± SD with significance determined by two-way ANOVA, * $p< 0.05$, n =17-35. ANOVA results: interaction: F (1, 99) = 6.206, $p = 0.0144$; Row factor (control vs *gcgr*$^{-/-}$): F (1, 99) = 1.600, $p < 0.2089$; Column factor (*pnoca* knockdown): F (1, 99) = 0.3003, $p = 0.5849$.

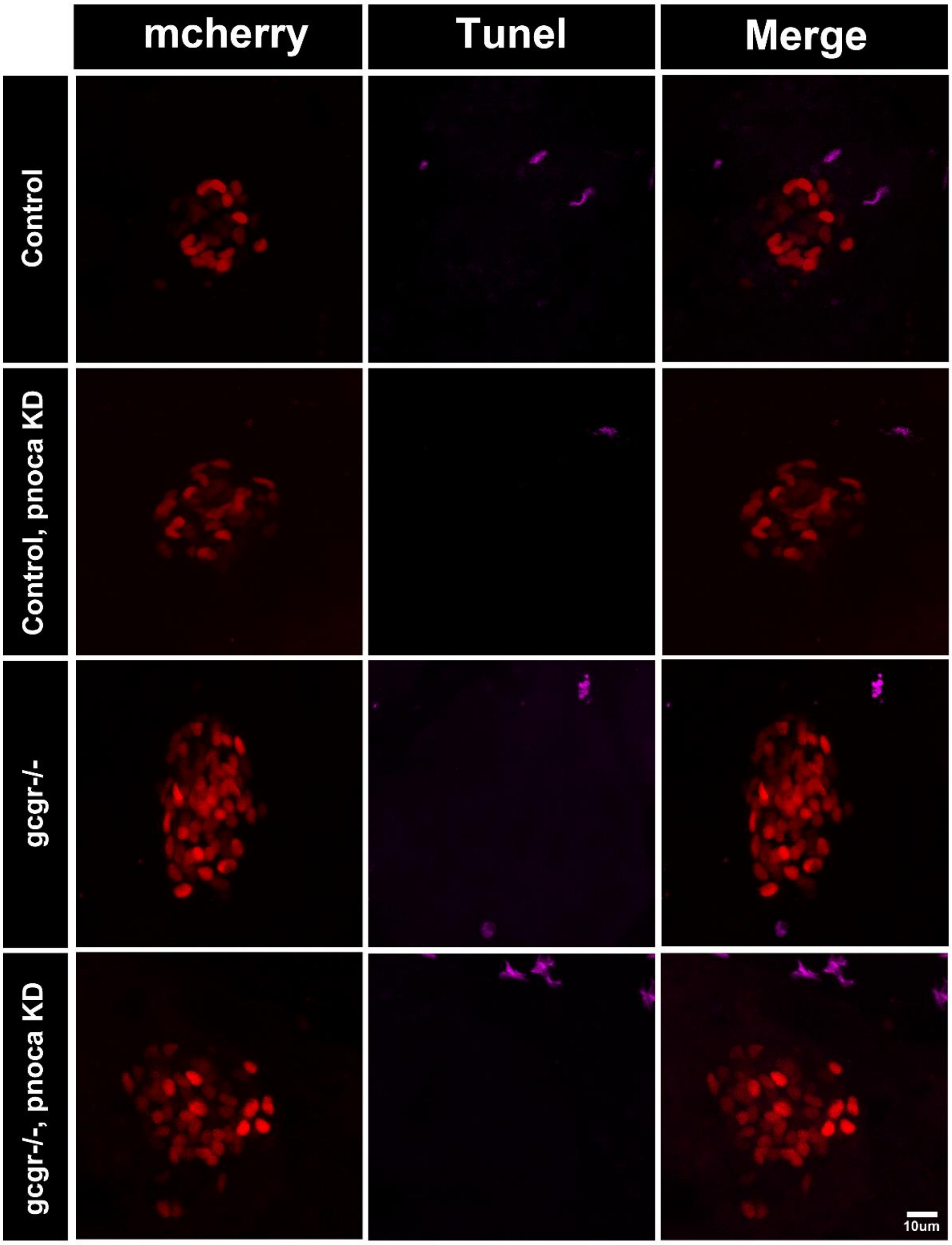

Fig S8, Knockdown of *pnoca* did not induce the apoptosis in α cell of control and *gcgr*$^{-/-}$.

Cell apoptosis in the *pnoca* knockdown larvae of control *Tg(gcga:H2BmCherry)* and *gcgr*$^{-/-}$*;Tg(gcga:H2BmCherry)* zebrafish groups were detected using a TUNEL apoptosis detection kit at 7dpf. Representative images of TUNEL labeling are shown, with Tunel^{+} cells representing the apoptotic cells indicated by magenta; there is no co-localization of magenta and mCherry in any group, n=7-12.

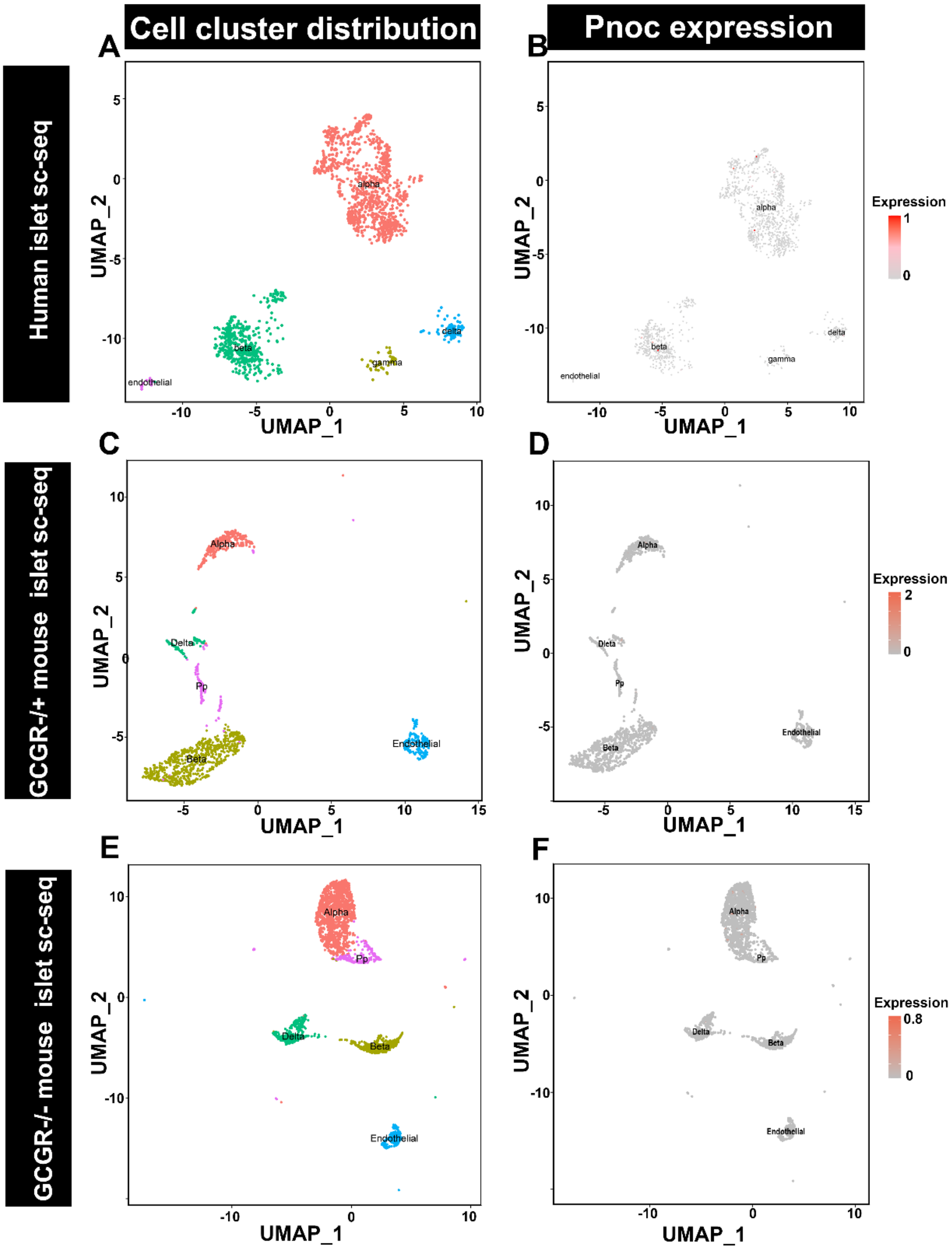

Figure S9, Expression profile of *Pnoc* in human and mouse endocrine cells. The analysis was based on the islet single-cell RNA-seq data obtained from NCBI GEO database (human GSE81547; mouse *Gcgr*$^{-/+}$ and *Gcgr-/-* GSE142233, in which *Gcgr*$^{-/+}$ was used as the control group). The endocrine cells type clusters distribution are indicated (**A**, **C** and **E**). The *Pnoc* expression levels are indicated in **B**, **D** and **F**.

The cell plots are based on the cell cluster shown in A, C and E respectively. The color scale ranges gray to red corresponding to expression levels low to high.
